# Supplementary material for: Framework-based qualitative analysis of free responses of Large Language Models: Algorithmic fidelity
Source: PLoS One. 2024 Mar 12;19(3):e0300024. doi: 10.1371/journal.pone.0300024 (PMC10931535; doi:10.1371/journal.pone.0300024)
Supplement: S1 Table — Belief statements were aligned based on their similarity when comparing human and silicon responses. ‘Rank’ refers to how frequently a domain was mentioned by humans and silicon participants, assessed separately for these groups. Instances, where beliefs are repeated, suggest that human participants expressed more detailed perspectives. For example, while humans might provide three distinct statements about how “Physical activity exacerbates my symptoms”—citing (1) fatigue, (2) a tight chest, and (3) heavy limbs—a silicon participant might simply state that “Physical activity worsens my symptoms”. (PDF) [file pone.0300024.s001.pdf]

Human				Silicon	
domain or construct	belief statement	rank	rank	belief statement	domain or construct
**Goals**		**1**	**6**		**Goals**
	**Enablers**			**Enablers**	
goal priority	Engaging in physical activity is a priority for me			Being physically active is a priority for me	goal priority
goal outcome	I engage in physical activity to be able to get on with life without help from others			Engaging in regular physical activity is important for maintaining my independence as I age	goal (outcome)
goal behavioural	I already engage in as much physical activity as I am able to			I try to stay active, despite my health conditions	goal (behaviour)
-	-			I'm staying physically active to ensure that I am taking care of my health	goal (outcome)
-	-			I engage in physical activity to prevent another heart attack	goal (outcome)
	**Barriers**			**Barriers**	
goal behavioural	I have integrated an adequate amount of physical activity into my life			I try to maintain an active lifestyle within the limitations of my condition	goal (behavioural)
goal conflict	Engaging in physical activity is not a priority for me			I get caught up in my daily responsibilities and it's hard to make time for physical activity	goal (behavioural) conflict
**Beliefs about Consequences**		**2**	**1**		**Beliefs about Consequences**
	**Enablers**			**Enablers**	
positive outcome expectancy (general health)	Physical activity improves my general health			I believe that staying active is crucial for maintaining my overall health and well-being, both physically and mentally	Positive outcome expectancy (general health)
positive outcome expectancy (mood)	I engage in physical activity because it makes me feel more cheerful			Physical activity can improve my mood	positive outcome expectancy (mood)
positive outcome expectancy (heart condition)	Physical activity improves the condition of my heart			Physical activity improves my cardiovascular health	positive outcome expectancy (cardiovascular health)
-	-			Physical activity helps me manage my heart failure symptoms	positive outcome expectancy (HF management)
-	-			Physical activity helps me manage rheumatoid arthritis symptoms	positive outcome expectancy (RA management)
-	-			Physical activity helps me to maintain my mobility and balance, and to prevent falls	positive outcome expectancy (mobility)
	**Barriers**			**Barriers**	
negative outcome expectancy (breathlessness)	Physical activity brings on my symptoms (e.g., breathlessness)			Physical activity makes my symptoms worse	negative outcome expectancy (symptoms HF)
risk perception (heart)	Physical activity is dangerous because it puts my heart under strain			I have to be more cautious and limit my activities to avoid putting too much strain on my heart	risk perception (heart)
-				I make sure that the activities I engage in are safe for me	risk perception (safety of exercise)
-				The main barrier is the risk of overexerting myself and making my condition worse	risk perception (health conditions)
negative outcome expectancy (fatigue)	Physical activity brings on my symptoms (i.e., fatigue)			Physical activity makes my symptoms worse	negative outcome expectancy (symptoms HF)
negative outcome expectancy (oedema)	Physical activity brings on my symptoms (i.e., heavy legs and arms)			Physical activity makes my symptoms worse	negative outcome expectancy (symptoms HF)
negative outcome expectancy (tight chest)	Physical activity brings on my symptoms (i.e., tight chest)			Physical activity makes my symptoms worse	negative outcome expectancy (symptoms HF)
lack of positive expectancy	Physical activity does not bring any benefits for me			-	
-				Pain and discomfort caused by physical activity as a result of my health conditions discourages me from being physically active	negative outcome expectancy (symptoms other health conditions)

Human				Silicon	
domain or construct	belief statement	rank	rank	belief statement	domain or construct
**Environmental context and resources**		**3**	**5**		**Environmental context and resources**
	**Enablers**			**Enablers**	
equipment	Equipment (bike; treadmill) helps me in being active			My home is also equipped with a stationary bike, which I can use in case the weather is bad or if I'm feeling particularly tired	equipment
implantable device (enabler)	Having an implantable device reassures me when engaging in physical activity			My implantable cardiac device gives me the confidence to stay active	implantable cardiac device (enabler)
group programmes	Group programmes help me in being physically active			Programs or resources available in my community help me with my physical activity	exercise programmes
treatment (enabler)	My heart failure treatment (i.e., medication) helps me in engaging in physical activity			-	
health-related event (enabler)	My physical activity levels increased since health-related event			-	
facilities	Facilities (e.g., local council) help me in being physically active			My home is designed for ease of movement and access, which helps me maintain my independence and ability to engage in my daily activities	facilities
local environment (enabler)	My local environment is a useful resource for my physical activity engagement			My environment provides me with many opportunities for physical activity	environment (enabler)
major life event (enabler)	My physical activity levels increased since a major life event			-	
	**Barriers**			**Barriers**	
health-related event (barrier)	My physical activity levels decreased since health-related event			-	
local environment (barrier)	My local environment limits me in engaging in physical activity (incline (hills); crowds; traffic; pollution)			My home and neighborhood environment can limit my physical activity options	environment (barrier)
treatment (barrier)	My heart failure treatment (i.e., medication) prevents me from engaging in physical activity			-	
implantable device (barrier)	My implantable device can harm me if I engage in physical activity			I avoid activities that put too much strain on my implantable cardiac device	implantable cardiac device (barrier)
major life event (barrier)	My physical activity levels decreased since a major life event			-	
-	-			I consider the weather, if it's too hot or too cold, it can make it harder for me to engage in physical activity	weather (barrier)
-	-			The area where I live makes it harder for me to access public transportation and other facilities that can promote physical activity like parks, gyms, or community centers	lack of facilities (barrier)
**Beliefs about capabilities**		**4**	**3**		**Beliefs about capabilities**
	**Enablers**			**Enablers**	
self-efficacy (Enabler)	I am confident in my ability to engage in physical activity			I am able to engage in light and moderate physical activity	self-efficacy (Enabler)
	**Barriers**			**Barriers**	
lack of self-efficacy (barrier, comorbidity)	Illnesses other than heart failure limit my ability to engage in physical activity			Fatigue and pain caused by my health conditions can be an obstacle to being physically active	perceived symptoms/self-efficacy
lack of self-efficacy (barrier, older age)	My limitations to engaging in physical activity is part of getting older			My physical activity is limited by my older age which causes lack of energy and can make it hard for me to be active	self-efficacy (older age)
lack of self-efficacy (barrier, HF symptoms)	Symptoms of my heart failure (e.g., breathlessness, tight chest, fatigue, swollen legs) limit my ability to engage in physical activity			My health conditions can make being physically active very difficult for me	self-efficacy (health conditions)
-	-			Before I developed heart failure, I was more physically active than I am now	self-efficacy (heart failure)
-	-			I find some types of physical activity too hard due to my health condition (e.g., strenuous activities, bending, kneeling)	self-efficacy (health conditions)
-	-			I also get tired easily and sometimes it's difficult to find the energy to move around	perceived exertion
-	-			A flare-up of my heart failure symptoms, such as fatigue and shortness of breath, limits my physical activity	perceived symptoms

Human				Silicon	
**domain or construct**	**belief statement**	**rank**	**rank**	**belief statement**	**domain or construct**
**Social influences**		**5**	**4**		**Social influences**
	**Enablers**			**Enablers**	
health professional advice	I engage in physical activity because a health professional (e.g., GP, consultant, nurse, physiotherapist) has advised me to do so			I make sure to follow my doctor's advice and guidance to maintain my health and well-being, and that includes being physically active	clinical advice
-	-			I find it helpful to have a partner or a friend to exercise with, as it keeps me motivated and accountable	social support (practical, companionship)
health professional advice (reassurance)	Having reassurance from a health professional that physical activity is safe encourages me to exercise			The advice and guidelines from my healthcare providers also help me to stay active in a safe way	clinical advice (reassurance)
social support (companionship, self-efficacy)	I would exercise with others if their level matched my capability			-	-
social support (emotional)	People who are important to me encourage me to be physically active			My friends and family have been helpful in encouraging me to be physically active	social support (emotional)
social norm	I am physically active because everyone I know closely is physically active			-	-
social contract, social support (instrumental, action planning)	Making plans with others encourages me to engage in physical activity			I find it helpful to have a partner or a friend to exercise with, as it keeps me motivated and accountable	social support (practical, companionship)
social learning	I know others my age who do not engage in physical activity much and their health deteriorated			-	-
-	-			Working with my healthcare team (e.g., physiotherapist) to find an appropriate exercise plan that is tailored to my health conditions and preferences and is safe for me is helpful	social support (practical), clinical advice, tailored exercise program from a physiotherapist
	**Barriers**			**Barriers**	
lack of social support (barrier)	People who are important to me discourage me from engaging in physical activity			My family and friends discourage me from being physically active	social influences (barrier)
health professional advice (barrier)	I limit my physical activity because a health professional (e.g., GP, consultant, nurse, physiotherapist) has advised me to not overdo it			Health professionals have advised me to listen to my body and make sure that any activity I do is safe for me	clinical advice (barrier)
-	-			My doctor has recommended that I avoid strenuous activities and not push myself too hard	clinical advice (barrier)
social support (instrumental, companionship)	I would engage in physical activity if it involved being with others			-	-
social support (instrumental)	I rely on other people to perform physical activity			-	-

domain or construct	Human belief statement	rank	rank	Silicon belief statement	domain or construct
**Behavioural regulation**		**6**	**2**		**Behavioural regulation**
	**Enablers**			**Enablers**	
implementation intention (pace)	I pace my physical activity to match my physical ability			I adjust my activity level accordingly to how I feel in a given moment	implementation intention
automaticity (habit)	Engaging in physical activity is something I do automatically			-	-
routine (habit)	I have a physical activity routine I follow			Having a regular routine and consistency in physical activity helps me to stay on track	habit
implementation intention (weather)	When weather is bad, I engage in physical activity indoors			If it's too hot, too cold, or raining, I exercise indoors	implementation intention
compulsion (habit)	I engage in physical activity compulsively			I try to make physical activity a part of my daily routine, so it becomes a habit	habit
self-monitoring (enabler)	I monitor intensity and/or duration of physical activity to make sure I do enough			Tracking my progress helps me to stay motivated and focused	self-monitoring
action planning	I know when and where I will engage in physical activity over the next week			I also find that having a plan and schedule for my physical activity helps me to stay on track	action planning
-	-			I try to find activities that I enjoy doing, which helps me to stay engaged and motivated	implementation intention
-	-			I try to make physical activity fun and enjoyable and easy to remember, so it becomes a habit	habit
-	-			Setting specific, realistic achievable goals for physical activity helps me in becoming more physically active	goal setting
-	-			Sometimes I also feel tired or lazy and I just don't want to go out and be active, but I try to overcome that by reminding myself of the benefits of staying active	implementation intention
-	-			I find safe and comfortable places where I can engage in physical activity when I am faced with a challenging environment	implementation intention
-	-			I incorporate a variety of activities to keep it interesting and focus on activities that are enjoyable	implementation intention
-	-			I gradually increase the intensity or duration of activity, which can help make it more manageable	implementation intention
-	-			I set reminders for myself to stay active (scheduling a walk in the calendar, setting an alarm)	reminders
-	-			I make a plan for how I will achieve my physical activity goals	action planning
-	-			I have strategies for clearing my mind so I can focus on physical activity	implementation intention
-	-			When I have a specific plan for my physical activity, it's easier for me to stay motivated	action planning
-	-			I try to minimize distractions and create a conducive environment to stay focused and motivated in being physically active	minimize distraction
	**Barriers**			**Barriers**	
self-monitoring (barrier)	I monitor the intensity and duration of physical activity to make sure I do not overdo it			I monitor physical activity to make sure I do not overdo it	self-monitoring (behavior)
-	-			I have learned how to pace myself and listen to my body to avoid overexerting myself	implementation intention
-	-			My sedentary lifestyle can make it hard to break the habit of being inactive	habit discontinuity
-	-			I monitor my heart rate and breathing during physical activity, which helps me to stay within safe limits and avoid overexerting myself	self-monitoring (heart rate and breathing)
-	-			I have to monitor my condition more carefully because of my cardiac implantable device	symptom monitoring

Human				Silicon	
**domain or construct**	**belief statement**	**rank**	**rank**	**belief statement**	**domain or construct**
**SPR**		**7**	**14**		**SPR**
	**Enablers**			**Enablers**	
identity and past behaviour (enabler)	I have always been an active person			Physical activity is a big part of who I am as a person	perceived identity (enabler)
identity (enabler)	Being physically active is a big part of my life			-	-
	**Barriers**			**Barriers**	
identity (barrier)	I am not a particularly active person			Physical activity is not a defining aspect of my identity	perceived identity (barrier)
identity (physical capacity, barrier)	I am not an athletic person			-	-
**Reinforcement**		**8**	**7**		**Reinforcement**
	**Enablers**			**Enablers**	
intrinsic reward (behaviour)	I enjoy engaging in physical activity			Physical activity is something that I enjoy doing	perceived intrinsic reward
intrinsic reward	I exercise because I feel much better when I have done so			When I feel good, I am more likely to do physical activity, which in turns improves my mood and well-being	positive feedback from behavior (?)
extrinsic reward (sense of accomplishment)	I feel pleased with myself when I have engaged in some physical activity			The sense of accomplishment I get from being active keeps me motivated to stay active	intrinsic (reward)
extrinsic reward (praise, social network)	I engage in physical activity because people close to me are pleased with me when I do				
extrinsic reward (praise, health professional)	I engage in physical activity because my doctor or other health professionals are pleased with me when I do			My doctor is usually the person that praises me for sticking to my physical activities and maintaining a healthy lifestyle	extrinsic reward (praise)
	**Barriers**			**Barriers**	
extrinsic punishment (symptoms)	Negative consequences of PA (pain; getting out of breath) discourage me				
lack of intrinsic reward	Physical activity is not an enjoyment, it is an obligation				
				I get discouraged if I don't see immediate results	negative reward (?)
				I may feel discouraged if I am not able to complete a physical activity due to my health conditions	negative reward (?)
**Emotion**		**9**	**9**		**Emotion**
	**Enablers**			**Enablers**	
				When I am feeling good and upbeat, I tend to have more energy and motivation to engage in physical activity	mood (enabler)
	**Barriers**			**Barriers**	
fear	I am too worried about the consequences of being active			What distracts me from being physically active is my fear of overexerting myself and causing harm to my heart	fear
mood (barrier)	I have to be in a mood to exercise				
mood (boredom, barrier)	I only engage in physical activity when I am bored				
low mood (barrier)	Low mood prevents me from engaging in physical activity			When I am feeling down, I may find it harder to be motivated to do physical activity	mood (barrier)
				It's also common to forget to be active when you're experiencing negative moods or emotions	negative emotions
				If I have too much on my mind, stress or anxiety, it can be hard to focus on physical activity and make it difficult to get started	stress and anxiety
				On bad days, I may feel frustrated or discouraged about my limitations and have less motivation to be active	frustration

	Human			Silicon	
**domain or construct**	**belief statement**	**rank**	**rank**	**belief statement**	**domain or construct**
**Knowledge**		**10**	**12**		**Knowledge**
	**Enablers**			**Enablers**	
knowledge (safety, enabler)	I know how much physical activity I can safely do			I have educated myself on how to engage in physical activity safely and appropriately for my condition	knowledge (safety, enabler)
				I understand that physical activity is essential for maintaining my overall health and well-being, especially with my heart condition	knowledge (benefits, enabler)
				I know how to manage my symptoms and stay active and healthy	knowledge (symptom management, enabler)
				I may feel some discomfort or aches during or after physical activity, but I consider it to be a normal part of exercise	knowledge (somatic, enabler)
				In the past, I have developed a certain level of knowledge and understanding about my arthritis and heart failure, which has helped me make informed decisions about my physical activity	knowledge (disease, enabler)
	**Barriers**			**Barriers**	
lack of knowledge (safety, barrier)	I don't know whether physical activity is safe for me			I do not know how much physical activity and what activities are safe for me	lack knowledge (safety, barrier)
				I would like to know more about how to best maintain my activity level as I age	lack knowledge (exercise techniques, barrier) (?)
**Optimism**		**11**	**8**		**Optimism**
	**Enablers**			**Enablers**	
dispositional optimism	I am optimistic by nature				
situational optimism (future behaviour)	I am optimistic that I will engage in physical activity in the near future				
hope	I hope to remain physically active				
situational optimism (ability)	I am optimistic about my ability to engage in physical activity			I am optimistic that I will be able to continue to stay active and maintain a consistent physical activity routine	optimism about future ability
				I always try to keep my options open and consider new ways to stay active	new ways to stay active
				I try to stay positive and focus on the benefits of physical activity	positive attitude
				I believe that with the right mindset and approach, it is possible to maintain an active lifestyle despite any challenges	positive attitude
				I try to focus on what I can do, rather than dwelling on what I can't do	positive attitude
				Having a positive attitude towards physical activity helps me in being physically active	positive attitude to physical activity
	**Barriers**			**Barriers**	
situational pessimism (ability)	I am fairly pessimistic about my ability to engage in physical activity in the future			I have to be realistic about my health condition and my limitations, and that can affect my optimism/expectations about physical activity at times	pessimistic expectation
**MADP**		**12**	**11**		**MADP**
	**Enablers**			**Enablers**	
memory (enabler)	I do not normally forget about my scheduled exercise (eg., class, group activities, gym)				
	**Barriers**			**Barriers**	
cognitive overload (barrier)	Engaging in physical activity requires a lot of thought and planning				
forgetfulness	I sometimes forget to follow my physical activity routine			I sometimes forget to be physically active	forgetfulness
symptoms hypervigilence (barrier)	I am vigilant to my heart rate and chest sensations when I am exercising			Before deciding to engage in physical activity, I listen to my body and pay attention to my symptoms, such as pain, fatigue, breathlessness or any other discomfort	symptom hypervigilance
				I have to be mindful of any pain or discomfort that I might feel during physical activity, and I have to stop if it becomes too much	symptom hypervigilance
				I get easily distracted by other things, such as TV or reading	attention/distraction

Human domain or construct	Human belief statement	rank	rank	Silicon belief statement	Silicon domain or construct
**Intention**		**13**	**13**		**Intention**
	**Enablers**			**Enablers**	
intention (enabler)	I try to engage in some form of physical activity every week			I will try to maintain my current level of physical activity and do what I can within my limits	intention (maintain, within physical ability limit)
				I am willing to make an effort to increase my physical activity levels	intention (to increase)
				I plan to continue staying as active as possible, within the limits set by my doctor.	intention (maintain, within doctor advice)
	**Barriers**			**Barriers**	
lack of intention (barrier)	I do not intend to be active for the sake of being active				
**Skills**		**14**	**10**		**Skills**
	**Enablers**			**Enablers**	
skills and training (enabler)	Being shown how to perform a form of physical activity helped me to become more physically active			I have learnt new types of exercise techniques that are safe for me given my health conditions	exercise techniques
				I have developed some skills and understanding in the past that have helped me to be physically active safely and effectively	skills (safety)
				I have learnt how to properly use any exercise equipment and how to perform exercises correctly to minimize the risk of injury	skills (equipment)
				In the past, I have developed the skill of listening to my body, which is important in being able to stay active without overdoing it	skills (or symptom hypervigilance?)
				I have learned some techniques to manage my arthritis pain, which has helped me to be more active	symptom management
	**Barriers**			**Barriers**	
lack of skills, gait, balance (barrier)	After I have been hospitalised, I had to get used to walking again (I had to learn)				
lack of skills and training (barrier)	I require training to be able to perform physical activity			In the future, as my health conditions change, I may need to learn new exercises or activities that are better suited to my abilities	lack exercise techniques
				I may also need to learn how to use new equipment or technology that can help me to stay active	lack skills (equipment)
				As for the future, I think that learning some new techniques and exercises that are specifically geared towards managing heart failure and arthritis	lack skills (symptom management)
				I might also need to learn how to monitor my heart rate, and how to avoid complications	lack skills (symptom hypervigilance)

Table 1: **Belief statements: Human (left) and Silicon (right)**. Belief statements were aligned based on their similarity when comparing human and silicon responses. The term **'Rank'** refers to how frequently a domain was mentioned by humans and silicon participants, assessed separately for these groups. Instances, where beliefs are repeated, suggest that human participants expressed more detailed perspectives. For example, while humans might provide three distinct statements about how "Physical activity exacerbates my symptoms" — citing (1) fatigue, (2) a tight chest, and (3) heavy limbs — a silicon participant might simply state that "Physical activity worsens my symptoms".
