# Supplementary material for: Framework-based qualitative analysis of free responses of Large Language Models: Algorithmic fidelity
Source: PLoS One. 2024 Mar 12;19(3):e0300024. doi: 10.1371/journal.pone.0300024 (PMC10931535; doi:10.1371/journal.pone.0300024)
Supplement: S1 File — (PDF) [file pone.0300024.s002.pdf]

S2. Sensitivity Analysis

Temperature

To assess the effect of a different temperature settings for LLM sampling, we repeated a subset of the interviews (n=6) using the "playground" interface and selected the following settings. We conducted a series of interviews with the GPT-3.5-turbo-instruct, (completion mode), simulating a conversation with a silicon participant named Linda, discussing the barriers and enablers to physical activity. Each version of Linda's response was generated with a distinct temperature setting: 1, 0.5, and 0, for each, the physically active (n=3) and sedentary lifestyle (n=3) mentioned in the backstory. In our examination of the interviews conducted with GPT-3.5-turbo-instruct, we focused on responses to the question "Can you please summarise what barriers you face in being physically active and what are the enablers?". Our interviews with GPT-3.5-turbo-instruct revealed a high degree of consistency in response patterns and depth when compared to GPT-3.5, and were also consistent across distinct temperature setting: 1.0, 0.5, and 0.0. Note that GPT-3.5-turbo-instruct and the customizable playground interface were not available during our initial study phase, hence why they were not used in the original set of interviews.

We did not observe any qualitative differences between these interviews and the original set. Illustrative examples are detailed below for the silicon participant Linda.

Linda, 77 years old, generally physically active, temperature 0.5, 400 tokens, date: 03/12/2023, GPT-3.5-turbo-instruct:

Well, some barriers I face include my health conditions, which can limit my mobility and energy levels. The weather can also be a barrier, especially in the winter months. But I have found that having a positive attitude and determination to stay active has been a great enabler for me. Having a supportive community and access to resources such as walking trails and home exercise equipment have also helped me stay physically active.

Linda, 77 years old, generally physically active, temperature 0, 400 tokens, date: 03/12/2023, GPT-3.5-turbo-instruct:

The main barrier I face is my health conditions, such as heart failure and rheumatoid arthritis. They can make it difficult to engage in physical activity at times. However, I have found ways to work around them, such as modifying my activities and listening to my body. The enablers for me are having a supportive community, finding activities that I enjoy, and staying motivated to improve my overall health and well-being.

Linda, 77 years old, generally physically active, temperature 1, 400 tokens, date: 03/12/2023, GPT-3.5-turbo-instruct:

Linda: Some of the barriers I face include my heart condition and arthritis, which make it difficult to do strenuous activities. And living in the countryside can also make it challenging to find opportunities for physical activity. However, some enablers for me include taking short walks every day, doing gentle exercises, and listening to my body to avoid overexertion.

GPT-4

To assess the effect of using a different underlying LLM model (GPT-4), we used the following settings, simulating the conversation with Linda again as described above. We used the chat mode within the "playground" interface, with the default system prompt "You are a helpful assistant" and temperature = 0.5.

We did not observe any qualitative differences in barriers or enablers between these interviews and the original set. There was one small difference in transcript style. GPT-4 very commonly includes descriptions of facial expressions or "stage directions" (e.g. "Linda: (Nods thoughtfully): ..."). Illustrative examples are detailed below for the silicon participant Linda.

Linda, 77 years old, generally physically active, temperature 0.5, 400 tokens, date: 03/02/2024, GPT-4:

Linda: (Nods thoughtfully) Well, my health conditions are the main barriers, dear. My heart failure limits how much activity I can do at a time, and my rheumatoid arthritis can make some movements painful. The weather can also be a barrier. If it's too cold or wet, it's hard to get outside for gardening or walking Daisy.

As for enablers, living in the countryside is a big one. I have plenty of space to garden and walk. My dog, Daisy, is another enabler. She needs her walks, and that motivates me to get up and move. And my family is very supportive. My daughter introduced me to chair yoga, and that's been a big help. Lastly, I'd say my own determination is an enabler. I want to stay as active as I can for as long as I can.
